# Outbreak of Pertussis, Kabul, Afghanistan

**DOI:** 10.3201/eid1407.071329

**Published:** 2008-07

**Authors:** Emmanuel Sagui, Lénaïck Ollivier, Tiphaine Gaillard, Fabrice Simon, Patrick Brisou, Philippe Puech, Alain Todesco

**Affiliations:** * Hôpital d’Instruction des Armées Laveran, Marseille, France; † Institut de Médecine Tropicale du Service de Santé des Armées, Marseille Armées, France; ‡ Hôpital d’Instruction des Armées Saint Anne, Toulon Naval, France; Etat Major 4, Marseille Armées

**Keywords:** whooping cough, Bordetella pertussis, Afghanistan, Kabul, respiratory tract disease, military personnel, letter

**To** **the Editor:** Infectious diseases are the main cause of illness for armed forces in conflict (*1*), resulting in decreases in operational efficiency. The International Security Assistance Force (ISAF) in Afghanistan is a multinational force operating under the auspices of the North Atlantic Treaty Organization (NATO). As part of ISAF, French troops operate in Kabul and its surroundings, within a 70-km radius. French medical facilities consist of a French field hospital and a primary care center. The facilities support 4,000 soldiers, 1,048 of whom are French.

Troop disease, including acute respiratory disease (ARD), is routinely monitored through French Army and NATO surveillance systems. We report an outbreak of ARD in the multinational force in which pertussis cases were identified by using laboratory tests and epidemiologic criteria.

In November 2006, a substantial increase of ARD was detected in soldiers of different nationalities (Figure), with a 10-fold increase among French troops at week 51. Patients with persistent cough or dyspnea were referred to the field hospital, in a nonrandomized manner, and those with a 2-week history of cough underwent serologic tests. Samples were sent to France and were analyzed at Hôpital Saint Anne, Toulon, France. Immunoglobulin (Ig) G antibodies to *Bordetella pertussis* antigens (pertussis toxin, filamentous hemagglutinin, and adenylcyclase) were determined by a Western blot assay (MarDx Diagnostics, Carlsbad, CA, USA). Recent infection was diagnosed by finding high levels of antibodies to pertussis toxin compared to results for standardized positive and negative samples, in concurrence with the fact that no soldier had been vaccinated against pertussis after childhood. IgG and IgA antibodies to *Chlamydia pneumoniae* were determined by a semiquantitative method that assessed samples’ absorbance value in optical density (SeroCP Quant IgG and Quant IgA, Savyon Diangostics, Ashdod, Israel). Recent infection to *Mycoplasma pneumoniae* was assessed by detecting IgM antibodies with a specific enzyme immunoassay (Platelia *Mycoplasma pneumoniae*, Biorad, Hercules, CA, USA) and by using a semiquantitative method to detect IgM and IgG antibodies with patented gelatin particles sensitized with cell membrane components of *M. pneumoniae* (Serodia Myco II, Fujirebio, Malvern, PA, USA). *Coxiella burnetii* infection was assessed by indirect immunofluorescence assay (*Coxiella burnetii* Spot IF, bioMérieux, Marcy l’Etoile, France).

**Figure:**
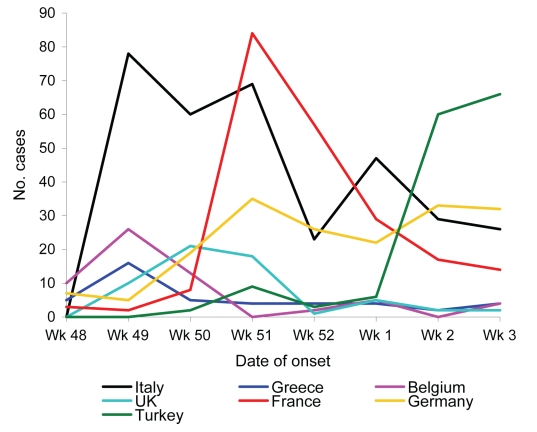
Number of acute respiratory diseases cases, according to troop nationality. UK, United Kingdom. A color version of this figure is available online: www.cdc.gov/EID/content/14/7/1173-G.htm

Statistical analysis was performed with Epi Info v3.4 software package (Centers for Disease Control [CDC], Atlanta, GA, USA). Quantitative variables were compared by using the Kruskall-Wallis test.

From the third week of December 2006 until the third week of January 2007, 209 French soldiers sought treatment at the French medical facilities for stereotyped acute febrile respiratory infection, which represents a cumulative attack rate of 20% on clinical grounds. Thirty-nine French soldiers and 10 non-French soldiers or local civilian workers were then referred to the field hospital. All patients had a 24-h history of fever >38.5°C and nonspecific ear, nose, and throat symptoms, mainly a sore throat. Cough was unremarkable, without whoops. Fourteen of the 49 patients were hospitalized for severe dyspnea. The difference in median age was not significant between inpatients (26 [range 20–57] years) and outpatients (36 [range 21–53] years, p = 0.15).

Twenty-seven blood samples were taken, 24 from French troops, 2 from British troops, and 1 from Polish patients. Six patients, including 3 French soldiers, had recent pertussis. No significant difference in age was found between patients with pertussis and those with non–pertussis ARD (36 [range 27–51] versus 33 [range 20–63] years; p = 0.39). No pertussis patient had been vaccinated against the illness since childhood.

One patient had evidence of recent infection with *M. pneumoniae*, and another with *C. pneumoniae*. No recent infection involved *C. burnetii*. All patients with ARD had a favorable outcome.

This outbreak of ARD among troops in Afghanistan highlights the importance of nontraumatic illness in wartime when military field conditions enhance exposure to, and incidence of, endemic diseases. Although our study did not include systematic laboratory confirmation for all cases of ARD in soldiers because of field conditions, this outbreak was mainly due to pertussis: most cases were defined by a cough lasting >2 wk, took place in an outbreak setting, and were (for 6 patients) confirmed by laboratory tests. CDC requirements were followed to ascertain confirmed cases (*2*). This outbreak also involved British troops; after the 2 cases we described, 2 additional serologically confirmed cases and 1 probable confirmed case were discovered among symptomatic British returnees (*3*). Pertussis, which remains endemic in developing countries (*4*), was reported in northeastern Afghanistan in 2002 (*5*), but was never biologically ascertained nor reported in Kabul.

This outbreak elicits 3 main questions. First, how can ARD transmission be stopped under field conditions? Besides prophylactic antibiotherapy, isolation of suspected case-patients is not achievable because of limited number of beds in medical facilities and high-person density in barracks and dining halls. To minimize transmission, patients and caregivers should wear masks.

Second, what prophylactic antibiotherapy should be given? We recommend a 3-day regimen of azithromycin because it is as efficient as erythromycin in preventing spread of pertussis (*6*), targets most intracellular bacteria involved in ARD, and offers the best compliance (*7*).

Finally, should soldiers be vaccinated against pertussis for overseas campaigns? In France, no booster vaccination is given after 13 years of age (*8*). Because acellular vaccines do not ensure immunity for >6 years (*5*), no French soldier has immunity to pertussis. We therefore advocate booster vaccination before overseas campaigns. Pertussis vaccination is widely available in combination with vaccination against, at minimum, diphtheria and tetanus, but these combination vaccines can only be administered once in an adult’s life and only 2 years after previous vaccination against diphtheria or tetanus. Monovalent vaccines against pertussis must be made more widely available for multinational troops in field conditions.
